# Supplementary material for: ACTH(6–9)PGP Peptide Protects SH-SY5Y Cells from H2O2, tert-Butyl Hydroperoxide, and Cyanide Cytotoxicity via Stimulation of Proliferation and Induction of Prosurvival-Related Genes
Source: Molecules. 2021 Mar 26;26(7):1878. doi: 10.3390/molecules26071878 (PMC8036943; doi:10.3390/molecules26071878)
Supplement: Supplementary file 1 [file molecules-26-01878-s001.pdf]

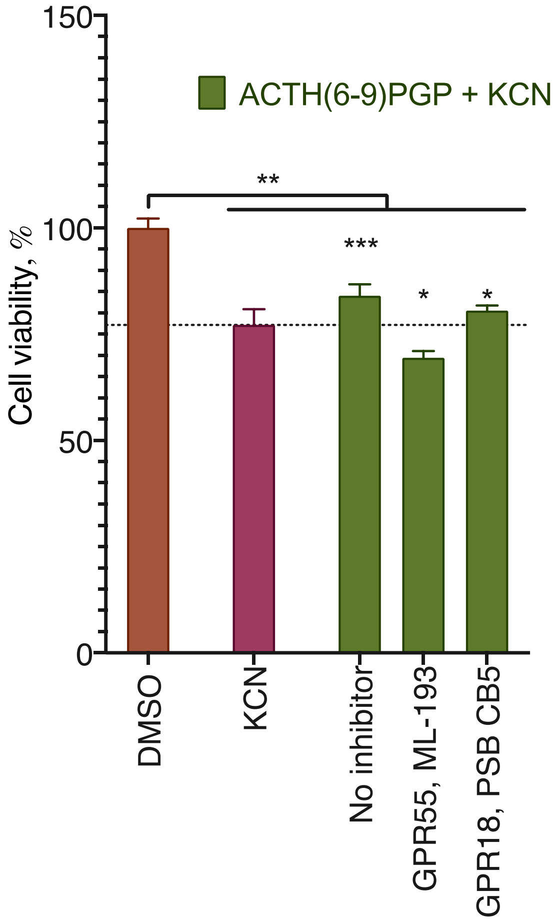

Figure S1. GPR55 and GPR18 participation in ACTH(6–9)PGP protection against the KCN cytotoxicity for the SH-SY5Y cells. Inhibitors ML-193 (2 μM) and PSB C5 (0.5 μM) were added 1 h before the KCN, and then together with KCN (950 μM) and peptide (50 μM). The cells were incubated with the inhibitors, KCN, and peptide for 24 h. MTT assay data, mean±standard error. *, statistically significant difference from the KCN+peptide without any inhibitor; **, statistically significant difference from the untreated control, ***, statistically significant difference from KCN alone, $p \leq 0.05$, ANOVA with the Tukey post-test, N = 3 experiments.

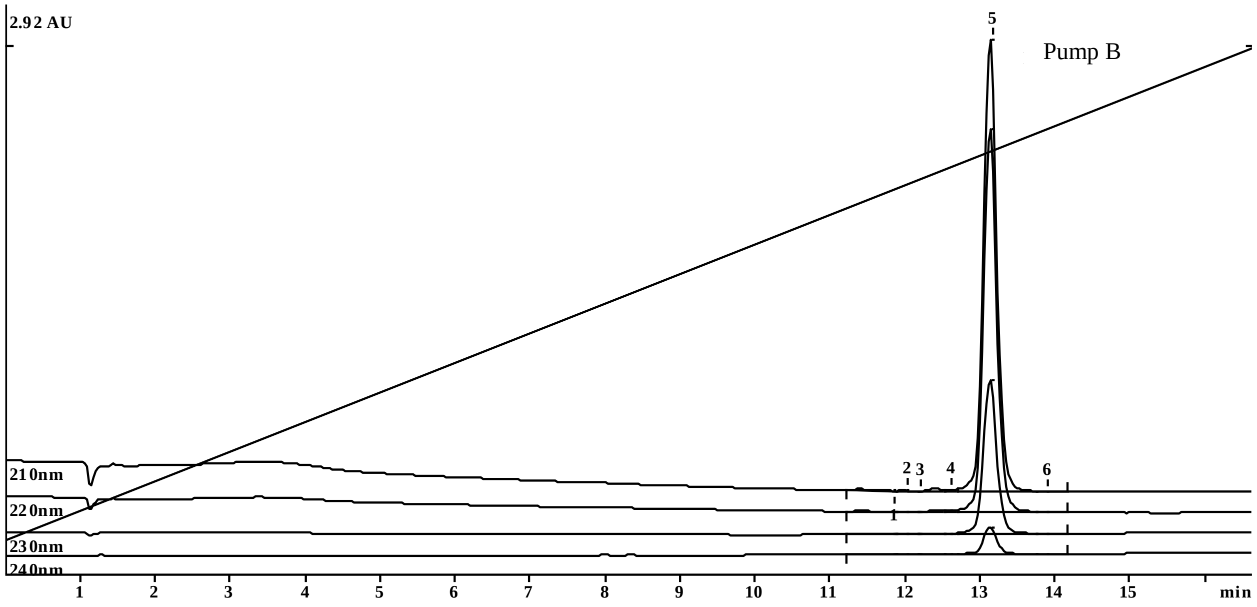

Figure S2. The homogeneity of the peptide was checked using high performance liquid chromatography (HPLC), and the structure was confirmed by mass spectrometry. The specific rotation of the plane of polarization of the light of the obtained peptides was determined on a Polarimeter A. Krüss Optronic GmbH P8000-T, t = 20 °C, in methanol, C = 1. The synthesized peptide was characterized by mass spectrometry on a ThermoElectron LCQ Advantage MAX instrument. Chromatographic conditions for the analysis of the peptide. Chromatograph: MiliChrome-A-02; Column: Prontosil 120-5C18aq, 2 × 75 mm. Eluent A: 0.2M $LiClO_4$ + 5mM $HClO_4$. Eluent B: methanol. Flow rate: 150 μL / min. Wavelength set: 210, 220, 230, 240 nm. Retention time = 13.14 min. Purity – 98,989. $[\alpha]_D^{20}$ = - 48^0

Table. The shape of the gradient for separating the synthesized peptides.

Time	%B
0	5
16.5	80

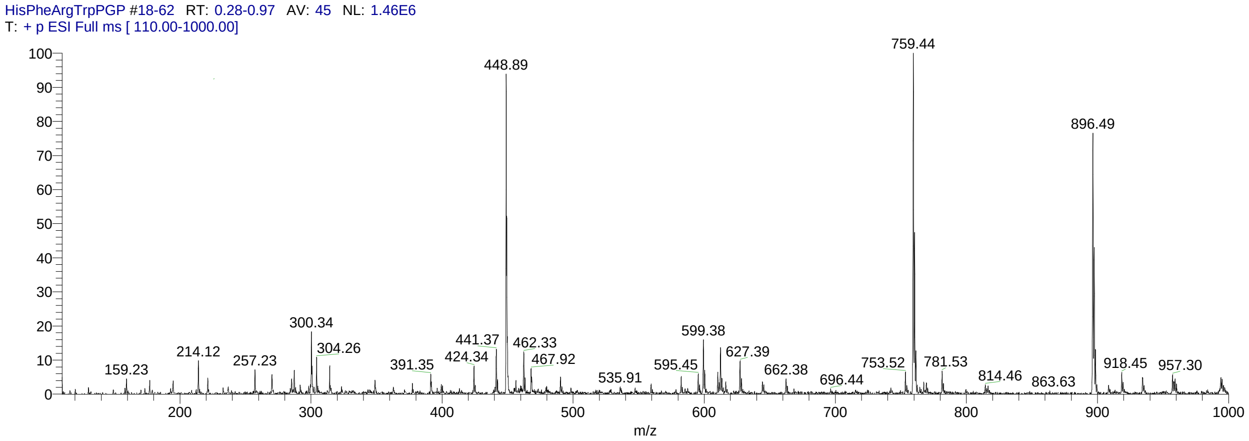

Figure S3. Full mass spectrum 896.4 = $[M + H]^+$ 448.9 = $[M/2+2H]^{2+}$. 759.4 = [M + H − His].

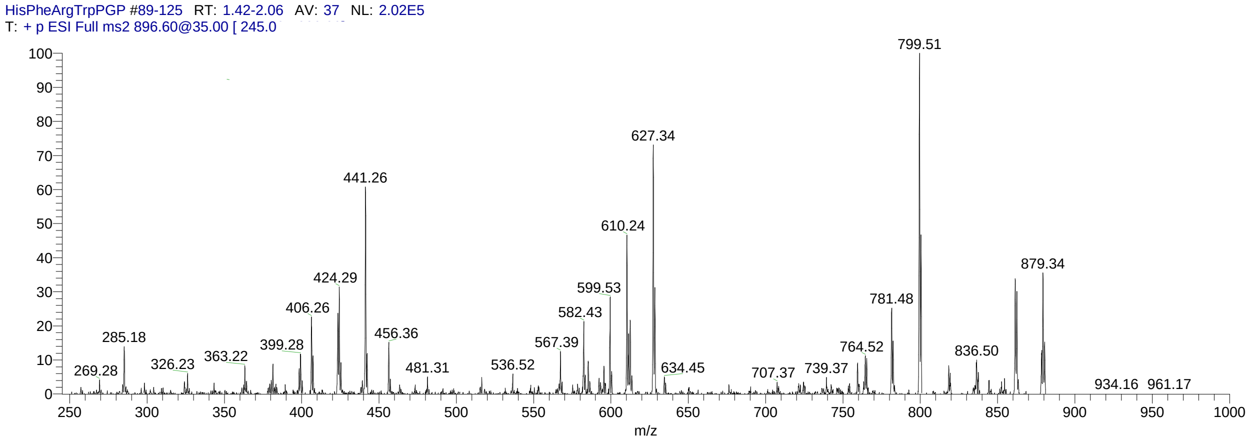

Figure S4. Fragmentation of the ion with *m/z* 896.4 $[M + H]^+$ @35eV. 782 = b6(HisPheArgTrpPG) 627 = b4(HisPheArgTrp) 441 = b3(HisPheArg) 285 = b2(HisPhe)

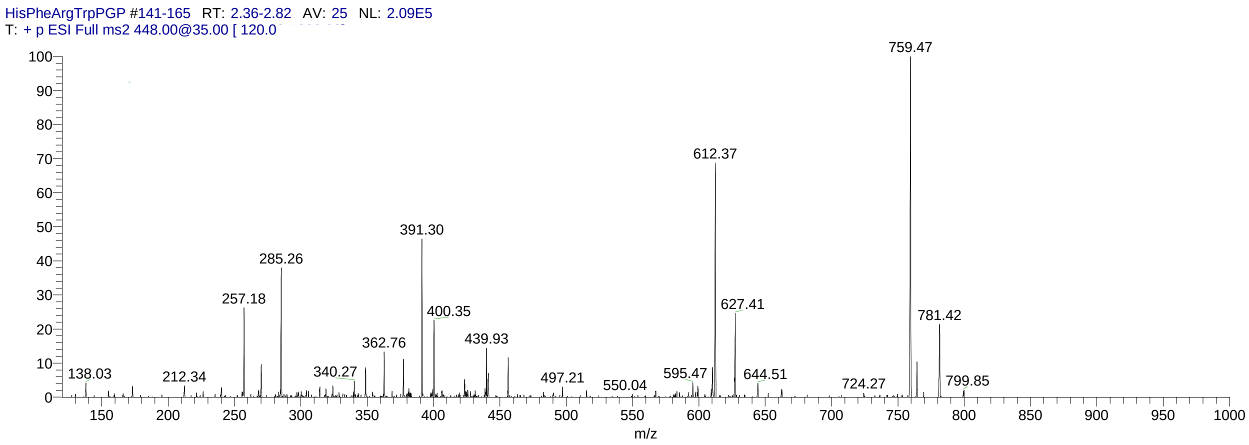

Figure S5. Fragmentation of the ion with *m*/*z* 408.8 $[M/2 + 2H]^{2+}$ @35eV. 759 = y6(PheArgTrpPGP) 612 = y5(ArgTrpPGP) 456 = y4(TrpPGP) 270 = y3(PGP) 173 = y2(GP) **781 = b6**(HisPheArgTrpPG) 724 = b5(HisPheArgTrpP) 627 = b4(HisPheArgTrp) 441 = b3(HisPheArg) 285 = b2(HisPhe)
